# Protein content and amino acid composition in the diet of Danish vegans: a cross-sectional study

**DOI:** 10.1186/s40795-023-00793-y

**Published:** 2023-11-15

**Authors:** Margit D. Aaslyng, Astrid Bøgebjerg Dam, Iben Lykke Petersen, Tenna Christoffersen

**Affiliations:** 1 University College Absalon, Nutrition and Health, Sdr. Stationsvej 30, Slagelse, 4200 Denmark; 2 Department of Food Science, University of Copenhagen, Rolighedsvej 26, Frederiksberg C, 1958 Denmark

**Keywords:** Vegan, Plant-based diet, Protein quality, Nutrition, Three-day dietary records

## Abstract

**Background:**

A growing proportion of the population are replacing their dietary animal protein with plant protein. A particular example of this trend is the vegan diet, which excludes all food items of animal origin. However, the DIAAS score for individual plant proteins is generally lower than that of animal proteins due to an unbalanced amino acid composition and lower bioavailability. Care must therefore be taken to meet the nutritional recommendations in the daily food intake.

**Methods:**

A three-day dietary food record was carried out by 40 Danish vegans in a cross-sectional study. The data were analysed, with particular emphasis on protein requirements and the essential amino acid composition of the diet.

**Results:**

The protein recommendations were met on all three days by 60% of the participants. In contrast, 18% did not meet the protein recommendations on any of the three days and 7% met the recommendations on only one of the days. Lysine was the most limiting amino acid (only 50% met the recommendations every day) followed by the sulphur-containing amino acids (recommendations met by 67.5%), leucine and valine (recommendations met by 70%). Combining both the amount of protein and the intake of the essential amino acids showed that less than half of the participants met the recommendations on all three days (47.5%) and 35% did not meet the recommendations on any days or on one day only.

**Conclusion:**

In conclusion, our study showed that many of the participants in the present study failed to meet the daily protein intake requirements, both on single days and on all three days. Furthermore, the food intake was found to have an inadequate amino acid composition and was particularly limited by the essential amino acids lysine, the sulphur-containing amino acids, and leucine and valine. This could be ascribed to the fact that only a limited number of protein sources were consumed during a day.

## Background

More and more people are changing to a diet low in meat or even avoiding meat altogether. There are many reasons for doing this, including health, the environment, animal welfare and religion [1–3]. Veganism is a particular kind of meat avoidance, in that no products of animal origin are included in the diet [4]. A vegan diet has several positive aspects such as a high intake of dietary fibers and a superior fatty acid composition [1, 5]. In addition, it is well documented, that a plant-based diet both vegan and vegetarian has several health benefits with respect to cardiovascular diseases, type 2 diabetes, cancer and obesity [6–8]. However, even though health is one of the underlying reasons for choosing a vegan diet, a vegan diet can also lead to concerns over whether the diet is sufficiently balanced to meet the individual’s nutritional requirements, not just in terms of micronutrients [3, 9] but also in terms of protein intake and protein dietary quality [10]. Protein dietary quality implies a sufficient supply of essential amino acids and a high bioavailability [6, 8, 11]. It can be measured as the DIAAS score (Digestible Indispensable Amino Acid Score), with a DIAAS score of 100 indicating that the protein meets the body’s needs and is pronounced as excellent protein quality [8, 12].

Individual plant proteins have a lower dietary protein quality than animal proteins due to the presence of anti-nutritional compounds resulting in reduced digestibility and also due to an insufficient amino acid composition in which one or several of the essential amino acids do not meet human requirements [11, 13–15]. For example, the DIAAS score for pork is above 100, while it is below 75 for fava beans as reviewed by Herreman et al. [12]. To achieve a sufficient protein quality in a diet, all nine essential amino acids (EAA) need to be present in sufficient quantities in the diet eaten over the course of a day [8] and not necessarily in the individual proteins. Proteins from pulses, such as peas and chickpeas, are low in the sulphur-containing amino acids cysteine (Cys) and methionine (Met) as well as in tryptophan (Trp), while cereals, nuts and seeds such as flaxseed are low in lysine (Lys) [8, 11]. By combining different sources of protein in a vegan diet, a full amino acid profile can be achieved [7, 10, 14, 16], and when this is achieved, a plant-based diet can be as effective for muscle protein synthesis as an animal-based diet [17–20]. In contrast, other studies show that the circulating blood level of the essential amino acids Lys, Cys and Met has been reported to be lower in men who follow a vegan diet compared with a meat diet, indicating that the difference between the amino acid compositions in the diet is reflected in the blood [21].

Several studies have focused on the nutritional quality of a vegan diet from a theoretical perspective through analysing model meals or from different dietary record studies, as recently reviewed [22, 23]. However, these studies focus mainly on the energy content and the content of macro- and micronutrients, whereas only few studies discuss the protein dietary quality and then only through a theoretical meal design [14, 16]. Many of the studies that include dietary records show a significant difference between a vegetarian (ovo-lacto vegetarian) diet and a vegan diet in the intake of energy and protein. For example, in one study, the protein intake per day for vegans (n = 104) was lowest, while the protein intake for omnivores (n = 155) was highest with vegetarians, semi-vegetarians (self-declared) and pesco-vegetarians in between [5]. Another study with only men found that vegans (n = 269) had a lower intake of protein/kg BW compared to meat eaters (n = 3798) [24]. Even though the two studies show that the overall intake is lower in the vegan diet, all diets were within the recommendations for energy and protein intake. These studies do not include the intake of the individual essential amino acids, but only the overall protein intake. Two other studies investigated a vegan diet theoretically with regard to amino acid requirement. They demonstrated that, with a diet planned by professionals, it is possible to meet the recommended intake of EAAs [14, 16] even though it can be challenging to get a sufficient amount of Lys in particular, but also Val, Leu, Ileu, SAA and Thr in a daily vegan menu [16]. However, it still needs to be proven whether or not an actual vegan diet is sufficiently varied to meet the amino acid recommendations. Therefore, the aim of this study was to evaluate the intake of energy and protein dietary quality by examining the intake of protein and essential amino acids in the diet of a sample of Danish vegans using a three-day dietary record and relating it to the variation of the food items in the diet.

## Methods

### Recruitment

Participants following a vegan diet (i.e. a diet that excludes all food products of animal origin) were recruited through Facebook targeting local vegan societies (Vegan in (name of a town)… Facebook groups), and vegan exercising societies (vegans and exercise, Facebook groups) combined with the snowballing effect. Inclusion criteria were: a minimum of 16 years of age (meaning that they were independent of their parents’ consent), healthy, and recording data from all three days. Exclusion criteria were: metabolic disorders, regular intake of medicine, and food intolerance that could affect food intake. A total of 54 people were recruited, with 14 people dropping out because they failed to complete dietary records on all three days. Therefore, 40 people participated in the study. The participants comprised 36 females and four males, aged between 16 and 59 years (mean 32.3, std. dev. 12.7) from all over Denmark. Their Physical Activity Level (PAL) was between 1.3 and 1.8 (mean 1.53, std. dev. 0.12) (see Sect. 2.4 Data analysis). Their BMIs, calculated on the basis of self-reported weight and height, were between 18.1 and 50.2 (mean 24.8, std. 7.3). Informed consent for participation was obtained in advance before the participants had completed their dietary records. As an incentive, all participants received oral or written feedback on how to optimise their diet to meet the official recommendations as well as two tickets to the cinema, which was mentioned during the recruitment, but not given until they had completed their dietary records.

### Dietary record

Participants carried out a three-day dietary record on paper on two consecutive weekdays and one weekend day following the methodology by Ortega, Pérex-Rodrigo & López-Sobaler [25]. Before recording their diet, the participants received detailed written instructions on how to fill in the three-day dietary record. The dietary record included a separate page for each day describing a meal pattern (breakfast, snack, lunch, snack, dinner and snack) and physical activity throughout the day, and the participants filled in additional information regarding age, sex, weight and height. The participants were encouraged to continue their habitual dietary intake, dietary pattern and physical activity level. Moreover, they were encouraged to report their intake in grams or standardised household measures (e.g. spoon, glass, etc.), take pictures of the meals, send nutritional declarations from food products and report recipes and preparation methods. They were encouraged to be as specific as possible and to report their intake of foods and drinks directly after consumption. They were also instructed to return their dietary record by email when the three-day dietary recording was completed. After receiving the dietary log, oral feedback was given to each participant by a dietitian student.

### Data entry

When the dietary records were received, the data were entered in a web-based food calculation software program (Vitakost, Kolding, Denmark). The first recorded meal before noon was considered as breakfast, and the remaining meals were considered as snacks or main meals depending on the size of the meal.

For several of the food items, the amino acid composition was absent in Vitakost. In these cases, a recipe was found (e.g. for hummus) and entered manually. The nutritional content and amino acid composition were calculated on the basis of that recipe. If the amino acid composition of the ingredients was not available in the software (e.g. soy drink), the scientific literature was searched and the amino acid composition was entered manually. Protein supplements were also entered manually in Vitakost and included in the calculations, since they were part of a meal, for example a smoothie. In this way, the amino acid composition of the food items was added to Vitakost. The amino acid composition of at least 95% of each study participant’s protein intake had to be known before the participants could be included in the study.

Dietary supplements other than protein supplements were not included in the food calculations.

### Calculations

The percentage of covered energy, protein and amino acids was calculated as:

$$Percent\,covered\, = \,Intake*100/recommended\,intake$$

The recommended intake is described in Table 1. For protein, calculations were performed using both the WHO minimum requirement (0.66 g/kg BW) and the Nordic Nutrition Recommendation (0.8 g/kg BW).

**Table 1:** Recommended daily intake of dietary protein and amino acids [27, 29]. The dietary protein is in accordance with the WHO and the Nordic Nutrition Recommendation (NNR), while the amino acids are only in accordance with the WHO recommendation

	Recommended daily intake
Protein, g, WHO minimum	0.66 X Body Weight (kg)
Protein, g, NNR	0.8 X Body Weight (kg)
His, mg	10 X Body Weight (kg)
Ile, mg	20 X Body Weight (kg)
Leu, mg	39 X Body Weight (kg)
Lys, mg	30 X Body Weight (kg)
SAA^1^, mg	15 X Body Weight (kg)
AAA^2^, mg	25 X Body Weight (kg)
Thr, mg	15 X Body Weight (kg)
Trp, mg	4 X Body Weight (kg)
Val, mg	26 X Body Weight (kg)

^1^Sulphur-containing amino acids (Met and Cys)

^2^Aromatic amino acids (Phe and Tyr)

The energy requirement for each participant was based on estimates of Basal Metabolic Rate (BMR, taking sex into account) and Physical Activity Level (PAL).

$$Energy\,Expenditure\,\left( {EE} \right)\, = \,BMR*PAL$$

The BMR was calculated based on body weight (kg) and height (m) using the prediction equations given by Henry [26].

PAL was estimated based on the number of hours spent on different physical activity levels and converted into metabolic equivalent values using values from the Nordic Nutrition Recommendation [27] multiplied by the time spent on a corresponding activity divided by 24 h a day [28].

### Data analysis

A descriptive data analysis was performed using R (ver. 4.1.2) and RStudio (v1.4.1717).

The data for intake of energy and macronutrients are given as average, median, standard deviation and coefficient of variation (SD/Mean). The E-percent for protein, carbohydrate and fat is calculated as (g * kJ/g*100)/(total intake of kJ). The energy content for protein and carbohydrate is 17 kJ/g, while it is 37 kJ/g for fat.

For each participant, the number of days (0–3) on which the recommended intake of energy, protein and each of the essential amino acids was covered was calculated. The number of days on which both the protein intake and all the individual essential amino acids were covered, thus resulting in a fully balanced diet with respect to protein and amino acids, was also calculated.

The meal pattern was described in two ways: for each day, the source of the protein was described as a 0 (not present)/1 (present) variable for each of the protein categories. Only a protein intake above 2 g/meal was included. The source categories were: cereals excluding oats and rice (bread, pasta, etc.), oats, seeds, pulses (excluding peanuts, lentils and chickpeas), chickpeas, lentils, vegetables and fruits, nuts (including peanuts), pseudocereals (quinoa and hemp), rice and other items (yeast, protein powder in the meals, etc.). The number of days on which the most frequent food items (oat flakes, hummus, falafel and peanut butter) were consumed was counted.

## Results

Dietary intake of energy, protein and amino acid composition was analysed for 40 vegans based on a three-day dietary record. A considerable variation was seen among the participants in their intake of energy and macronutrients (Table 2). On average, they consumed 8.2 MJ per day, while the median was 7.4 MJ per day. However, the standard deviation was 3.3 MJ, indicating that some of the participants had a low energy intake while others had a high energy intake. In particular some of the participants had a high intake as shown by the difference between the average and the median. The variation in energy intake was reflected in all the macronutrients, with protein, carbohydrate and fat having a coefficient of variation (CV) of up to 53%.

**Table 2:** Average daily intake of energy, protein, carbohydrate and fat (n = 120 days, three-day record from 40 participants)

	Average	Median	SD	CV	E-percent
Energy, MJ	8.2	7.4	3.3	0.40	
Protein, g	66.3	61.9	29.5	0.44	14.2
Carbohydrate, g	243.2	232.4	101.0	0.42	53.3
Fat, g	70.5	62.1	37.6	0.53	30.5
Protein g/kg body weight	0.98	0.93	0.42	0.43	
Carbohydrate, g/kg body weight	3.59	3.49	1.44	0.40	
Fat, g/kg body weight	1.07	0.85	0.63	0.60	

The recommended energy intake was calculated on the basis of body weight and energy level (PAL score), whereas the recommended intake of protein and essential amino acids was calculated only on the basis of body weight. The PAL values were between 1.3 and 1.8 corresponding to a sedentary or light activity level [30]. The WHO recommends a minimum intake of protein of 0.66 g/kg BW per day [29]. However, in Denmark the recommendation is 0.8 g/kg BW partly to take into account the fact that the amino acid composition might not be optimal [27]. Both recommendations were met as an average over all records. Looking at the individual days, 60% of the participants had a sufficient protein intake to meet the WHO requirements on all three days, whereas only 50% met the NNR recommendations on all three days (Table 3). In comparison, 18% did not meet the recommended intake of protein on any of the days when taking the WHO recommendations into consideration, whereas this number increases to 30% when taking the NNR recommendations into consideration. Meeting the requirements on only one day showed the same pattern: 7% met the WHO recommendations and 5% met the NNR recommendations. In total, it seems that approx. 25% of all the participants were challenged in getting a sufficient amount of protein from their diet and meeting the WHO recommendations, with an even higher number (35%) not meeting the NNR recommendations. In contrast to the intake of protein, the recommended energy intake was not met on any of the days by more than half of the participants (55%) (Table 3), and only 10% met the recommended energy intake on all three days.

**Table 3:** Percentage of days on which the intake met the dietary recommendations for energy, protein (WHO: 0.66 g/kg BW, NNR: 0.8 g/kg BW) and essential amino acids (EAA, WHO recommendations) (n = 120 days, 40 participants each recording 3 days)

	0 days	1 day	2 days	3 days
Energy	55	10	25	10
Protein, WHO	18	7	15	60
Protein, NNR	30	5	15	50
His	2.5	0	17.5	80
Ile	7.5	7.5	10	75
Leu	12.5	10	7.5	70
Lys	15	17.5	17.5	50
SAA^1^	10	7.5	15	67.5
AAA^2^	0	0.0	7.5	92.5
Thr	2.5	15	7.5	75
Trp	0	2.5	5	92.5
Val	10	12.5	7.5	70
Protein and all EAAs, WHO	22.5	12.5	17.5	47.5

^1^Sulphur-containing amino acids (Met and Cys)

^2^Aromatic amino acids (Phe and Tyr)

There was variation in the intake of the individual EAAs. Almost all of the participants met the recommended intake on all three days both for the aromatic amino acids (AAA) and for Trp. In contrast, the recommended intake of Lys was only met on all three days by 50% of the participants followed by the sulphur-containing amino acids (SAA), which were met on all three days by 67.5% of the participants and Leu and Val which were met on all three days by 70% of the participants.

**Fig. 1:**
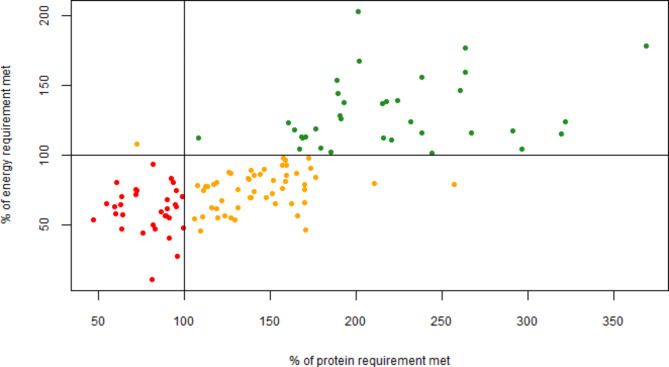
The relationship between meeting the recommended energy intake (y-axis) and meeting the recommended protein intake (based on WHO recommendations) (x-axis). Each spot represents one participant on one day. The green spots represent days with sufficient energy and protein intake, the yellow spots represent days on which the participants lacked either energy (lower right corner) or protein (upper left corner), and the red spots represent days on which the participants lacked both sufficient energy and protein

The relationship between the percentage of covered energy requirements and the percentage of covered protein requirements can be seen in Fig. 1. Only one participant on one day met the energy requirements without getting the necessary protein (upper left corner), indicating that protein intake is generally sufficient when energy intake is sufficient. In contrast, the recommendation for protein was met, though not for energy (lower right corner), on 52 days, corresponding to 43% of the days, and the opposite can therefore not be confirmed (i.e. a sufficient protein intake does not automatically result in a sufficient energy intake).

The frequency of intake of different food items indicate the variation in the participants’ diets (Table 4). A typical breakfast in this study consisted of oat flakes with some kind of vegan drink – soy, oat or pea. Some of the participants also consumed seeds and fruits for breakfast. In total, 75% of the participants ate oat flakes on at least one day (mostly for breakfast, but for some as a snack meal in the morning or afternoon), 57.5% ate them on at least two days and 42.5% ate them every day.

**Table 4:** Protein sources in analysed vegan diets (n = 120 days, 40 participants each recording food intake 3 days)

Protein source	Examples	% days out of 120^*^
Cereals (excluding oats and rice)	Bread, pasta, cakes	71.7
Oats	Oat flakes, oat porridge	60.8
Rice	White rice, brown rice	13.3
Pulses (excluding chickpeas, lentils and peanuts)	Spread, bean burger, beans, yoghurt, tofu	66.7
Chickpeas	Hummus, falafel	36.7
Lentils	Dahl, lentil spread	25.0
Vegetables, fruits	Banana, berries, cauliflower, potatoes	56.7
Nuts (including peanuts)	Peanut butter, almonds, hazelnuts	45.8
Other	Yeast flakes, protein powder	23.3
Seeds	Flaxseed, pumpkin seeds, chia seeds	22.5
Pseudocereals	Quinoa, hemp seeds	10.0

^*^three days dietary record from 40 participants

The lunch was slightly more varied and consisted mainly of wheat and rye bread, which is common in Denmark, with hummus or sometimes peanut butter, although some participants also ate a more complex lunch containing a lot of vegetables and sometimes beans or lentils. Hummus was part of the diet for more than half of the participants (55%) on at least one day, 30% ate hummus on at least two days and 10% ate hummus every day. Since 15% also ate falafel, chickpeas were one of the main pulses in the vegan diet and were eaten on 34.2% of all days. Although peanut butter is not a traditional part of the Danish diet, it was consumed by 35% of the participants, and 20% of the participants consumed peanut butter on at least two days.

The dinner differed considerably among the participants, with some having a very varied dinner containing a wide range of food items and others having a very conservative dinner, the same every day. In general, the participants prepared the dinner themselves, and some kind of convenience food, often pizza or burger, was consumed on only 14.2% of the days. This is in accordance with the study of Nebl et al. [31] in which the vegan participants consumed an average of only 16.6 g fast food per day.

As can be seen in Table 4, cereals and oats were very abundant in the vegan diet. This was followed by pulses and vegetables. In contrast, pseudocereals and rice did not make up a significant part of the protein intake. The protein typically came from three to five different sources per day (69% of the days). This indicates that, for most of the days, the diet was relatively uniform in terms of protein sources (Table 5).

**Table 5:** The availability of different protein sources in the vegan diet. To be included, at least 2 g of a protein source must be eaten per meal. The protein sources are defined in Table 4

Number of protein sources per day	1	2	3	4	5	6	7	8
Percentage of days	2	8	24	22	23	15	5	1

## Discussion

Changing the diet from animal based to plant based protein has several positive health effects [6]. However, since the DIAAS score for plant proteins is generally lower than the DIAAS score for animal proteins [8], care must be taken to meet the recommendation for dietary protein and amino acid intake. In this study, the actual food intake from 40 vegans over three days was measured using a three-day dietary record and the protein- and amino acid intake calculated.

An individual’s energy requirement is dependent on his/her sex, body weight and activity level, and this was calculated individually for each participant. In contrast, the protein recommendation was calculated as g/kg BW and was independent of sex and activity level. Some studies include only one sex, often men, [32] while others do not break down the results according to sex [33]. In studies that break down the results according to sex, the difference between the dietary groups (vegans, omnivores) is the same in the two sexes [34].

The energy intake was lower than recommended for most of the participants (55%) in our study (Table 3). However, with an average of 8.2 MJ (Table 2), it was at the same level as that of other studies reporting vegan diets ranging from 8.14 MJ/day [22] to 9.97 MJ/day [5]. Despite the low energy intake, the protein intake was to a larger degree sufficient and at 0.98 g/kg body weight it was on average above the level recommended by WHO (Table 2) which is in accordance with other studies which reported 0.94 g/ kg body weight [35], 1.0 g/kg body weight [36] and 1.01 g/kg body weight [37] and higher than that of another study which reported 0.64 g/kg body weight [38]. For the individual days, the protein intake was within the level recommended by the WHO on all three days for 60% of the participants (Table 3). This also means that, for 40% of the participants, their protein intake was below the recommended level on one or more days, and, for 25% of the participants, the protein intake was below the recommended level every day or on two out of three days. In comparison, Allès et al. [39] report that 27.3% of 789 vegan participants had a protein intake below the acceptable level while Waldmann et al. [35] report the same for 31.3% of the vegan males and 41.4% of the vegan females. In comparison, only 4% of the meat eaters and 15.3% of the vegetarians had a protein intake below the acceptable level in the study by Allès et al. [39]. Other studies state that the average protein intake is sufficient in a vegan diet [5, 36, 40]. For example, a study with 269 vegan males found that the average intake was 0.91 g/kg BW [24], although it did not investigate single days. To maintain body function, growth, and for females reproduction and lactation, protein intake needs to meet the recommendations each day [29]. It is therefore not sufficient just to look at the average consumption over several days or the average consumption for a population, as most studies do, but, as in our study, go into detail each day.

One concern in changing from animal to plant proteins in the diet is the protein dietary quality [3]. The quality of plant proteins is lower due to an unbalanced amino acid composition and a lower digestibility [11], and the circulating blood level of the EAAs in subjects who follow a vegan diet compared with subjects who follow an omnivore diet has been shown to be lower for Lys while different results have been seen for, Cys and Met [21]. Interestingly, the correlation between circulating plasma amino acids and the intake is low, as long as the intake is adequate [36, 38]. This confirms the results of other studies that show that plant protein can be as efficient as animal protein in muscle anabolism, as long as the amino acid composition is within the recommendation [17, 41]. In our study, Lys, followed by SAA, Leu and Val were the EAAs most often below the recommended level which corresponds to other studies [16, 21, 36].

Even though several studies state that a vegan diet overall can be healthier than an omnivore diet [5, 32], some studies point out that there can be drawbacks, for example in the immune system, to changing to a vegan diet [42] and also stress that the long-term effect of a vegan diet remains unknown [43]. We have shown that, even though almost half of the vegans in this study met the recommendations for protein and EAAs on all three days (47.5%), a significant number of them (35%) did not meet the protein and EAA requirements on most of the days, giving rise to nutritional concerns.

To ensure a balanced amino acid composition [19, 20] it is necessary to combine several different protein sources that complement each other [10]. The lower digestibility of plant proteins can be attributed to both the protein chemical structure and the interaction with other macronutrients, and the digestibility can be further impaired by the presence of antinutritional compounds [10, 11]. One way to compensate for the lower digestibility of plant proteins is to increase protein intake. However, half of the participants in our study did not meet the recommendations for protein intake on all three days. Another strategy is to enhance the digestibility by applying technological processing steps that can contribute to changes in the protein chemical structure and reduce the antinutritional components [3, 10, 11]. This might present a challenge, since focus groups have pointed out that there is a low level of trust among consumers regarding industrial products that they perceive as highly processed [44] and even though these consumers were omnivores, the same could be true for vegan consumers. It has also been stated that, unless the vegan diet is unrealistically uniform, the amino acid supply will be sufficient [45]. When considering the choice of food items, this study shows that the diet for many of the vegan participants was uniform. Cereals provided one of the main protein sources, with oats alone eaten on 60.8% of the days and other cereals eaten on 71.7% of the days (Table 4). Cereals are known to be low in Lys [46] in particular, and Lys is also the limiting amino acid in most of the diets (Table 3). This is further strengthened by the intake of different seeds in which Lys is also the limiting amino acid [11]. Peanut butter and nuts also tended to be popular among vegan consumers in this study, and they were eaten on almost half of the days. Peanut butter is high in energy, but, as with the cereals and seeds, Lys is the limiting amino acid [11]. Lys helps the body absorb calcium and also has an impact on the synthesis of collagen [47]. Lys deficiency has been demonstrated to impair amino acid metabolism and induce cellular imbalance [48]. This underlines the need to supplement the diet with other protein sources that are richer in Lys.

Rice is a cereal that is often eaten as the carbohydrate part of the meal, though the intake of rice was very low among the vegans in this study (Table 4). This might be because climate change is one of the main reasons many vegans avoid animal products [2], and the production of rice has a high climate impact [49].

Pulses were the second most frequently consumed protein source, followed by vegetables (Table 4). Other studies have reported the average intake per day measured in weight. In these studies vegetables and fruits are the most abundant food items with an intake having approximately ten times higher intake than that of legumes and three to five times higher than that of cereals and starchy food [31, 39]. However, this is problably because these food items are heavier than legumes and cereals and it does not indicate how often they are eaten. Chickpeas were the single most abundant food item, consumed as either hummus or falafel. Hummus is known to be a nutritious food item rich in protein, dietary fibres, micronutrients and different bioactive compounds, but, as with the other pulses, chickpeas need to be supplemented with other protein sources to achieve a complete amino acid profile [50]. In addition to chickpeas, different soy products such as yoghurt and soy drink made up most of the intake of pulses, although other types of beans were also included in the diet. Protein from pulses is known to have a low content of the SAAs [11], in particular, which is also reflected in the intake, since the intake of SAAs was the second most insufficient amino acid, achieving recommended levels on all days for only two thirds of the participants (Table 3). SAAs are involved in the synthesis of several key metabolites [47], and there are indications that a deficient intake of SAA might lead to neural disorders [51, 52]. Met and Cys are most abundant in the proteins of pseudocereals such as quinoa and hemp [11], and, as seen in Table 4, even though some of the participants supplemented their diet with hempseeds, neither of these pseudocereals was widespread in the diet.

Even though it is argued that it is possible to achieve a balanced amino acid intake by eating a varied diet containing different plant protein sources, Table 5 shows that the vegan participants’ diet in this study is mostly made up of three, four or five protein sources. Furthermore, the fact that some of the protein sources have the same limiting amino acids, in particular Lys, but also the SAAs, shows that the combination of different protein sources required in order to include all of the EAAs in a vegan diet in sufficient amounts, are not necessarily present today, since less than half of the participants met the recommended protein intake and all the amino acids on all three days (Table 3). The reason for the low variation in protein sources is unknown, but could be a lack of awareness, poor cooking skills or others.

Some studies have pointed out that many vegan consumers reject highly processed foods due to distrust [3, 44]. This was confirmed in our study, since convenience food made up only part of the diet on 14.2% of the days, often as some very well-known dishes such as pizza and burgers. Instead, the vegans made their own food, which for some of them resulted in uniform meals containing few protein sources, while others made very complex food from scratch containing several protein sources. This distrust is something of a dilemma, in that some kinds of processing, such as extrusion, can actually enhance protein digestibility [53]. Furthermore, the production of plant-based convenience foods also offers the possibility of combining protein sources to provide an adequate amino acid composition. The high intake of hummus and peanut butter further points to a need for greater variation in the processed food available on the market. A supply of different convenience food items with high nutritional quality and a high protein quality could help improve the quality of vegan diets. This should be accompanied with information regarding the health effect of these food items, to encourage the more sceptical vegans to include them in their diet rather than only benefiting those who are not wary of processed food.

According to Berrazaa et al. [10], a discussion has been ongoing on whether to increase the recommended protein intake in a vegan diet, since the protein dietary quality of plant protein is lower than that of animal protein. This idea has been rejected, since it is possible to combine different protein sources to achieve the required amino acid composition. Our study shows that a significant number of the participants did not achieve an adequate protein and amino acid intake in their diet every day and that complementary protein sources were not combined systematically. Furthermore, when taking into account lower protein bioavailability, it is necessary to provide more guidance on how to design an adequate vegan diet. Focus should also be aimed at developing mildly processed convenience food items that take into account improved protein quality.

The study has certain limitations. Nutritional value of a diet can be investigated by several methods. A three-day dietary record is a prospective open-ended assessment method used to record all food items consumed during the assessment period. The participant weighs all food items consumed on two weekdays and one weekend day and records detailed information such as recipes, preparation methods and brands [25]. Although this method is time-consuming and difficult for the participant to manage, it does have several advantages. First of all, the actual food intake is recorded, and the quantity of the intake is measured. This can subsequently be related to different databases of nutritional content, and the intake of macro- and micronutrients can be calculated. However, one drawback to using this method could be that the participant has an unusual dietary pattern during the three-day period due to the specific focus on the food. Another drawback is that the data are self-reported, and the intake might therefore be underestimated. Other methods used to estimate food intake include a 24-hour food interview in which the participant is interviewed about his or her consumption retrospectively and a food frequency questionnaire in which food items and portion sizes are systematically recorded. Since both the 24-hour food interview and the food frequency questionnaire are retrospective, they are challenged by the participant’s ability to recall his/hers intake, and, furthermore, the exact quantity of the food intake is not measured. In our study, we therefore decided to use the three-day dietary record to collect quantitative data on the actual intake on these days despite its limitations.

The amino acid content of many of the food items was not available in the databases. For some of the food items, the ingredients were present in the database, and we therefore calculated the amino acid content on the background of a recipe. For other food items, we searched the scientific literature to determine the amino acid composition. This introduced a degree of uncertainty regarding the intake of amino acids, but it was necessary to obtain enough information and it was more precise than only including the food items that were already present in the database.

Another limitation of the study is that the participants’ weight and activity level were self-reported. This means that their weights might have been underreported, which is a well-known problem [54], and therefore the calculated dietary needs might have been underestimated. In addition, more females than males participated in the study, which indicates that more females than males want to reduce their meat consumption [55] or that more females were willing to participate in this kind of a study. The recommended intake of protein, amino acids and energy was calculated on a personal level, and it is therefore not necessary to distinguish between sexes in the analysis. The age distribution was broad, which can also be seen as an advantage since the participants were representative of a broad section of the population.

During the recruitment, we did not ask the participants how long had followed a vegan diet, or about their motivations for this and whether they were trying to lose weight. These factors might have influenced their eating habits.

The sample number in this study was 40 people. They were not necessarily representative of all vegans in Denmark, as they were sampled using social media such as Facebook and the snowballing effect. Furthermore, the incentives of providing nutritional advice and offering tickets to the cinema were mentioned during recruitment and this might have further biased the sampling toward recruiting vegans who were not confident in their knowledge of what they should eat. When taking into account the time consumption and complexity of the three-day dietary record method, it must be expected that the participants represent the vegans who are most interested in diet and nutrition, since they are the most motivated. Although the number of people who follow a vegan diet in Denmark is unknown, a recent survey sample (n = 1005) using a sampling strategy to include as many vegan and vegetarian participants as possible, found 1% followed a vegan diet [56]. Relating this to the Danish population as a whole would mean that approximately 59,000 people in Denmark follow a vegan diet. However, since the survey was specifically aimed at this group of people, the actual number can be expected to be lower. According to the Danish Vegetarian Society, 45,000 Danes follow a vegan diet [57], which means that the 40 participants in this study represent between 0.09% and 0.07% of all Danes who follow a vegan diet.

In this study we focused on a vegan population and did not include other dietary groups. Therefore, part of the conclusion might also apply to other dietary groups though this was not within the scope of the study.

## Conclusion

In conclusion, our study showed that many vegan diets in the present survey failed to meet the daily protein intake requirements, both on single days and on all three days. Furthermore, the diets were particularly limited by the essential amino acids lysine, the sulphur-containing amino acids, leucine and valine. This could be ascribed to the fact that the diet was relatively uniform and only a limited number of protein sources were consumed during a day.

More guidance for vegans on how to design their diet to meet the recommendations for protein and amino acid intake is therefore necessary to overcome this challenge. Furthermore, there is a need for food items that contain different protein sources and further processed using gentle technology that can help enhance protein bioavailability. This should be accompanied with information about the health benefits of the food items aimed at the sceptical vegan consumers.

## Data Availability

The datasets used in the present study are available from the corresponding author on reasonable request.

## List of abbreviations

AAA: Aromatic amino acids
BMR: Basal Metabolic Rate
CV: Coefficient of variation
Cys: Cysteine
DIAAS: Digestible Indispensable Amino Acid Score
EAA: Essential amino acids
His: Histidine
Ile: Isoleucine
Leu: Leucine
Lys: Lysine
Met: Methionine
NNR: Nordic Nutrition Recommendation
PAL: Physical Activity Level
SAA: Sulphur-containing amino acids
Thr: Threonine
Trp: Tryptophan
Val: Valine
