# Correction: Protein content and amino acid composition in the diet of Danish vegans: a cross-sectional study

**DOI:** 10.1186/s40795-024-00866-6

**Published:** 2024-04-16

**Authors:** Margit D. Aaslyng, Astrid Bøgebjerg Dam, Iben Lykke Petersen, Tenna Christoffersen

**Affiliations:** 1 University College Absalon, Nutrition and Health, Sdr. Stationsvej 30, 4200 Slagelse, Denmark; 2 Department of Food Science, University of Copenhagen, Rolighedsvej 26, C 1958 Frederiksberg, Denmark

**Correction: BMC Nutr 9, 131 (2023)**.

10.1186/s40795-023-00793-y.

Following publication of original article [1], the authors would like to acknowledge that we have not been consistent in the use of the terms ‘recommended intake’ and ‘average requirement’. We would like to clarify, that the NNF protein norm is a recommended protein intake while the WHO protein and amino acids norms are average requirements. We apologize for the error and has corrected it below in **bold**.

In the abstract, the result section, the correct text should be:

The average protein **requirement** were met on all three days by 60% of the participants. In contrast, 18% did not meet **the requirement** on any of the three days, and 7% met the **requirement** on only one of the days. Lysine was the most limiting amino acid (only 50% met the **average requirement** every day) followed by the sulphur-containing amino acids **(average requirement** met by 67.5%), and leucine and valine (**average requirement** met by 70%). Combining both the amount of protein and the intake of the essential amino acids showed that less than half of the participants met the **average requirement** on all three days (47.5%) and 35% did not meet it on any days or on one day only.

Table 1. The table text should be: 

Table 1. **Average requirement (WHO, protein and amino acids)** and recommended intake (NNR, protein) [27, 29]. The dietary protein is in accordance with the WHO and the Nordic Nutrition Recommendation (NNR) while the amino acids are only in accordance with the WHO average requirement.

And the first calculation should be:

Percent covered = Intake*100/(recommended intake **or average requirement**).

Second paragraph in the data analysis should be:

For each participant, the number of days (0–3) on which the recommended intake**/average requirement** of energy, protein and each of the essential amino acids was covered was calculated.

Second and third paragraph in the results should be:

The recommended energy intake was calculated on the basis of body weight and energy level (PAL score), whereas **the average requirement**/recommended intake of protein and essential amino acids was calculated only on the basis of body weight. The PAL values were between 1.3 and 1.8, corresponding to a sedentary or light activity level [30]. The WHO **gives an average requirement** of protein of 0.66 g/kg BW per day [29]. However, in Denmark the recommendation is 0.8 g/kg BW, partly to take into account the fact that the amino acid composition might not be optimal [27]. Both **protein levels** were met as an average over the three days and for all participants. Looking at the individual days, 60% of the participants had a sufficient protein intake to meet the WHO requirements on all three days, whereas only 50% met the NNR recommendations on all three days (Table 3). In comparison, 18% did not meet the **average requirement** of protein on any of the days when taking the WHO **level** into consideration, whereas this number increases to 30% when taking the NNR recommendations into consideration. Meeting the requirements on only one day showed the same pattern: 7% met the WHO **average requirement** and 5% met the NNR recommendations. In total, it seems that approx. 25% of all the participants were challenged in getting a sufficient amount of protein from their diet and meeting the WHO **average requirement**, with an even higher number (35%) not meeting the NNR recommendations. In contrast to the intake of protein, the recommended energy intake was not met on any of the days by more than half of the participants (55%) (Table 3), and only 10% met the recommended energy intake on all three days.

There was variation in the intake of the individual EAAs. Almost all of the participants met the **average requirement** on all three days both for the aromatic amino acids (AAA) and for Trp. In contrast, the **average requirement** of Lys was only met on all three days by 50% of the participants followed by the sulphur-containing amino acids (SAA), which were met on all three days by 67.5% of the participants and Leu and Val which were met on all three days by 70% of the participants.

Table 3. Percentage of days on which the intake met the **average requirement/**dietary recommendations for energy, protein (WHO: 0.66 g/kg BW, NNR: 0.8 g/kg BW) and essential amino acids (EAA, WHO recommendations) (*n* = 120 days, 40 participants each recording 3 days).

Figure 1. The relationship between meeting the recommended energy intake (y-axis) and meeting the **average requirement of** protein (based on WHO) (x-axis). Each spot represents one participant on one day. The green spots represent days with sufficient energy and protein.

In the discussion, the first part of third paragraph should be:

The energy intake was lower than recommended for most of the participants (55%) in our study (Table 3). However, with an average of 8.2 MJ (Table 2), it was at the same level as that of other studies reporting vegan diets ranging from 8.14 MJ/day [22] to 9.97 MJ/day [5]. Despite the low energy intake, the protein intake was to a larger degree sufficient and at 0.98 g/kg body weight it was on average above the **average requirement stated by** WHO (Table 2) which is in accordance with other studies which reported 0.94 g/ kg body weight [35], 1.0 g/kg body weight [36] and 1.01 g/kg body weight [37] and higher than that of another study which reported 0.64 g/kg body weight [38]. For the individual days, the protein intake was within the level **of average requirement** by the WHO on all three days for 60% of the participants (Table 3). This also means that, for 40% of the participants, their protein intake was below the **average requirement** on one or more days, and, for 25% of the participants, the protein intake was below the **average requirement** every day or on two out of three days. In comparison, Allès et al. [39] report that 27.3% of 789 vegan participants had a protein intake below the acceptable level while Waldmann et al. [35] report the same for 31.3% of the vegan males and 41.4% of the vegan females.

The last sentence in paragraph 3 should be:

In our study, Lys, followed by SAA, Leu and Val were the EAAs most often below the **average requirement** which corresponds to other studies [16, 21, 36].

Paragraph 9 should be:

Even though it is argued that it is possible to achieve a balanced amino acid intake by eating a varied diet containing different plant protein sources, Table 5 shows that the vegan participants’ diet in this study is mostly made up of three, four or five protein sources. Furthermore, the fact that some of the protein sources have the same limiting amino acids, in particular Lys, but also the SAAs, shows that the combination of different protein sources required in order to include all of the EAAs in a vegan diet in sufficient amounts, are not necessarily present today, since less than half of the participants met the **average requirement of** protein intake and all the amino acids on all three days (Table 3). The reason for the low variation in protein sources is unknown, but could be a lack of awareness, poor cooking skills or others.
